# Supplementary material for: RNA sequencing identifies lung cancer lineage and facilitates drug repositioning
Source: PeerJ. 2024 Sep 24;12:e18159. doi: 10.7717/peerj.18159 (PMC11430167; doi:10.7717/peerj.18159)
Supplement: Supplemental Information 2 [file peerj-12-18159-s002.docx]

**SUPPLEMENTARY INFORMATION**


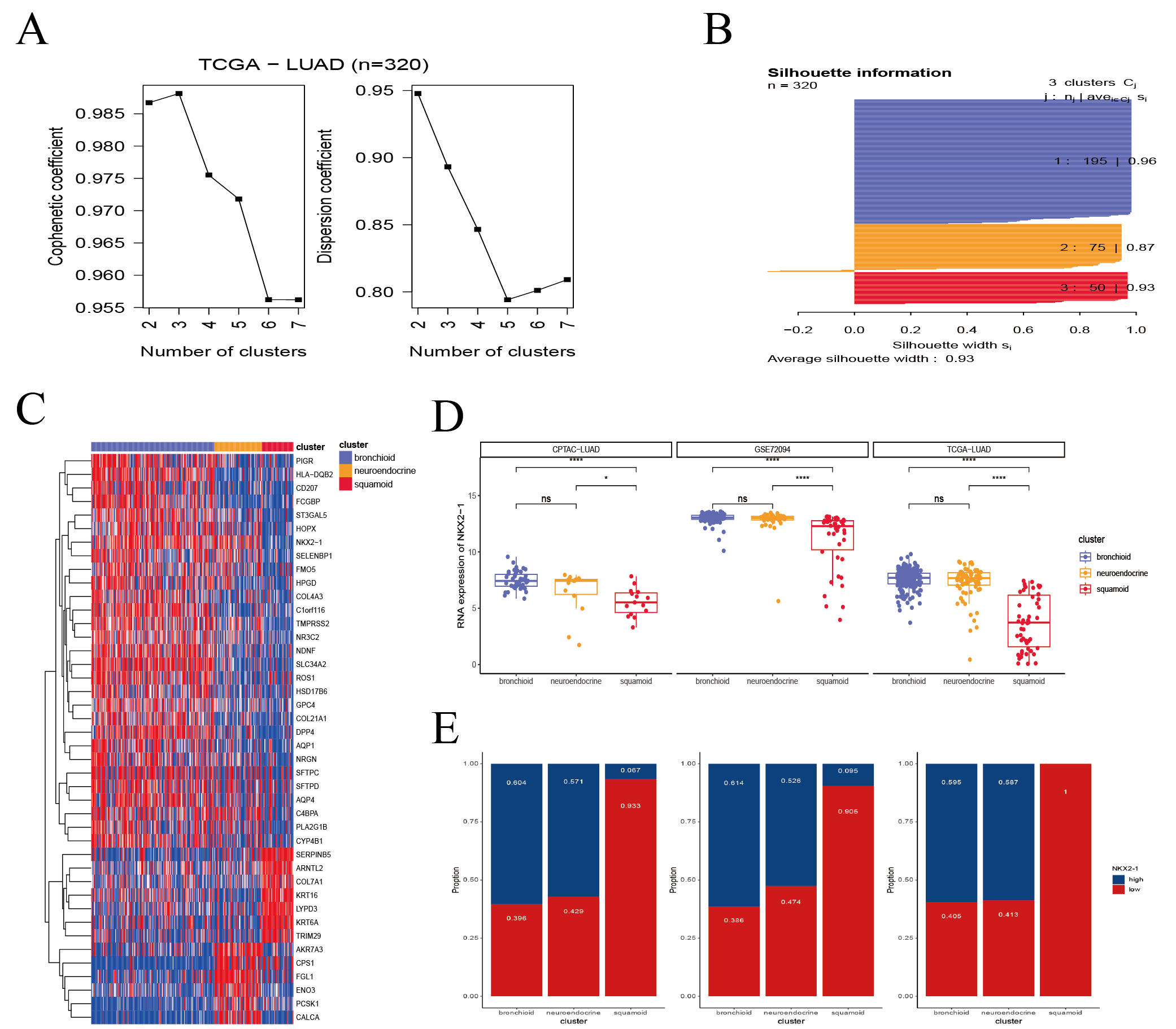


**Figure S1. Clustering analysis classified three prognostic clusters**

(A) Non-negative matrix factorization was performed in the stage IB–IIIA TCGA-LUAD cohort. Cophenetic coefficient and dispersion coefficient were shown for k = 2 to 7. (B) Silhouette information of stage IB–IIIA TCGA-LUAD for k = 3 was exhibited. (C) Heatmap showing two clusters using 42 candidate genes. The expression data was based on mean normalization. (D) Box plots showing the RNA expression of NKX2-1 among bronchioid, neuroendocrine and squamoid clusters. Comparison between groups using Wilcoxon-test. * and **** representing p<0.05, p<0.0001, respectively. (E) Bar plots represent the relationship between median defined NKX2-1 group and clusters.


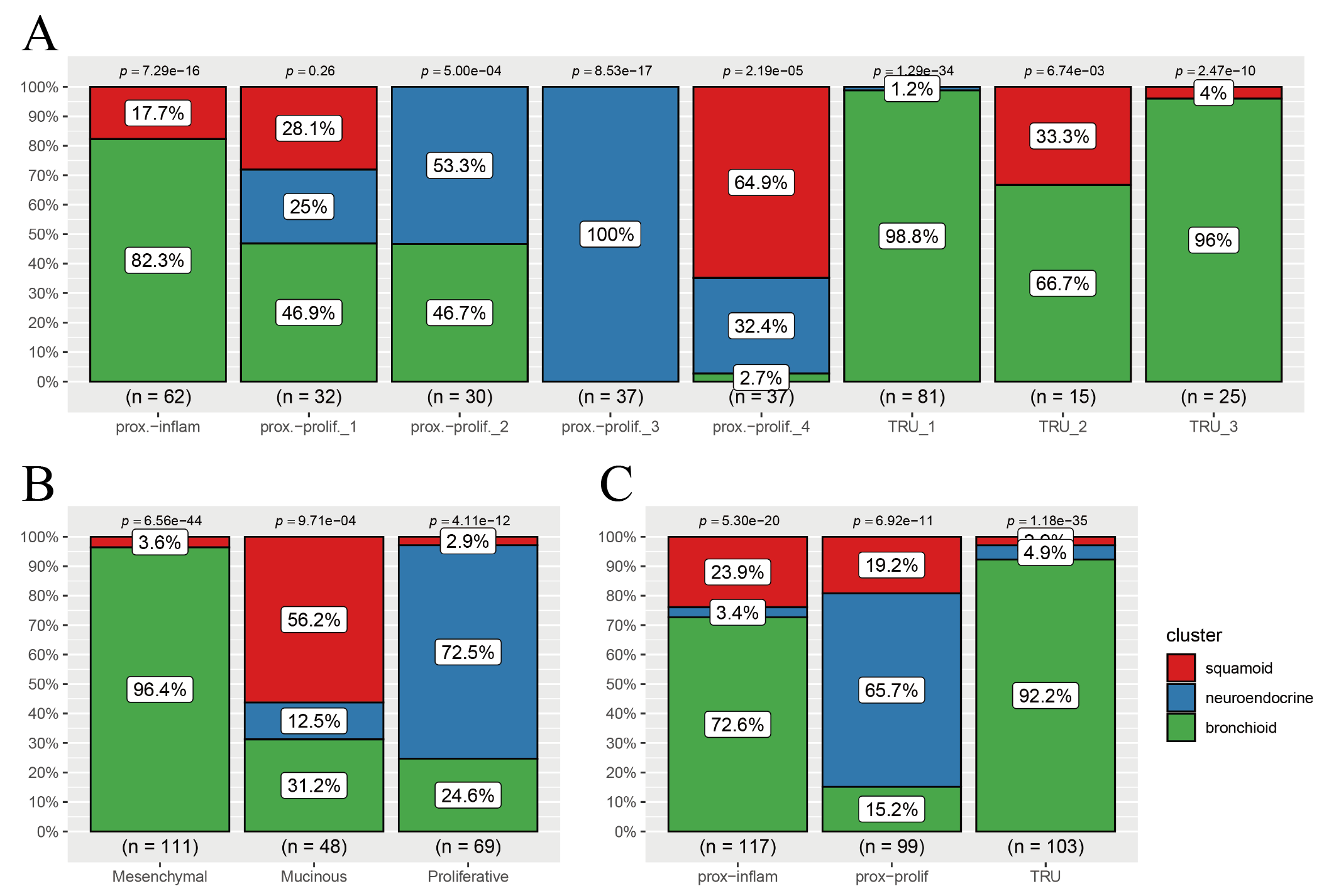


**Figure S2. Relationship between previous subtypes and three clusters**

Bar plots showing overlapping ratios between our clusters and (A) Wang et al, (B) Daemen et al, and (C) TCGA group subtypes in the TCGA-LUAD cohort.


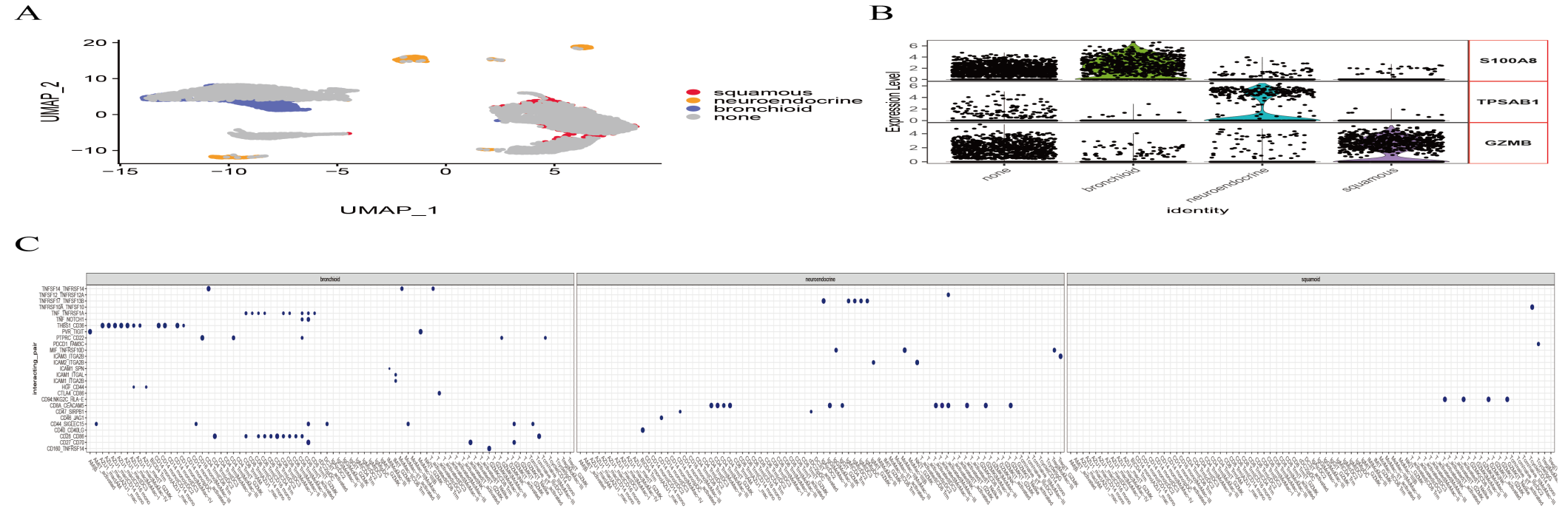


**Figure S3. Single-cell analysis among cluster-related cell subpopulation**

(A) Umap showing the relative distribution from bronchioid, neuroendocrine and squamoid predicted clusters. Note that “none” represents the low confidence or unrelated above clusters. (B) Boxplot plotting classical marker molecules among the above clusters. (C) Unique inter-cellular signals among the above clusters (The vertical axis represents ligand-receptor pairs, with larger dot representing lower p-value. Only incorporating mean expression greater than 1 and p-value less than 0.05).


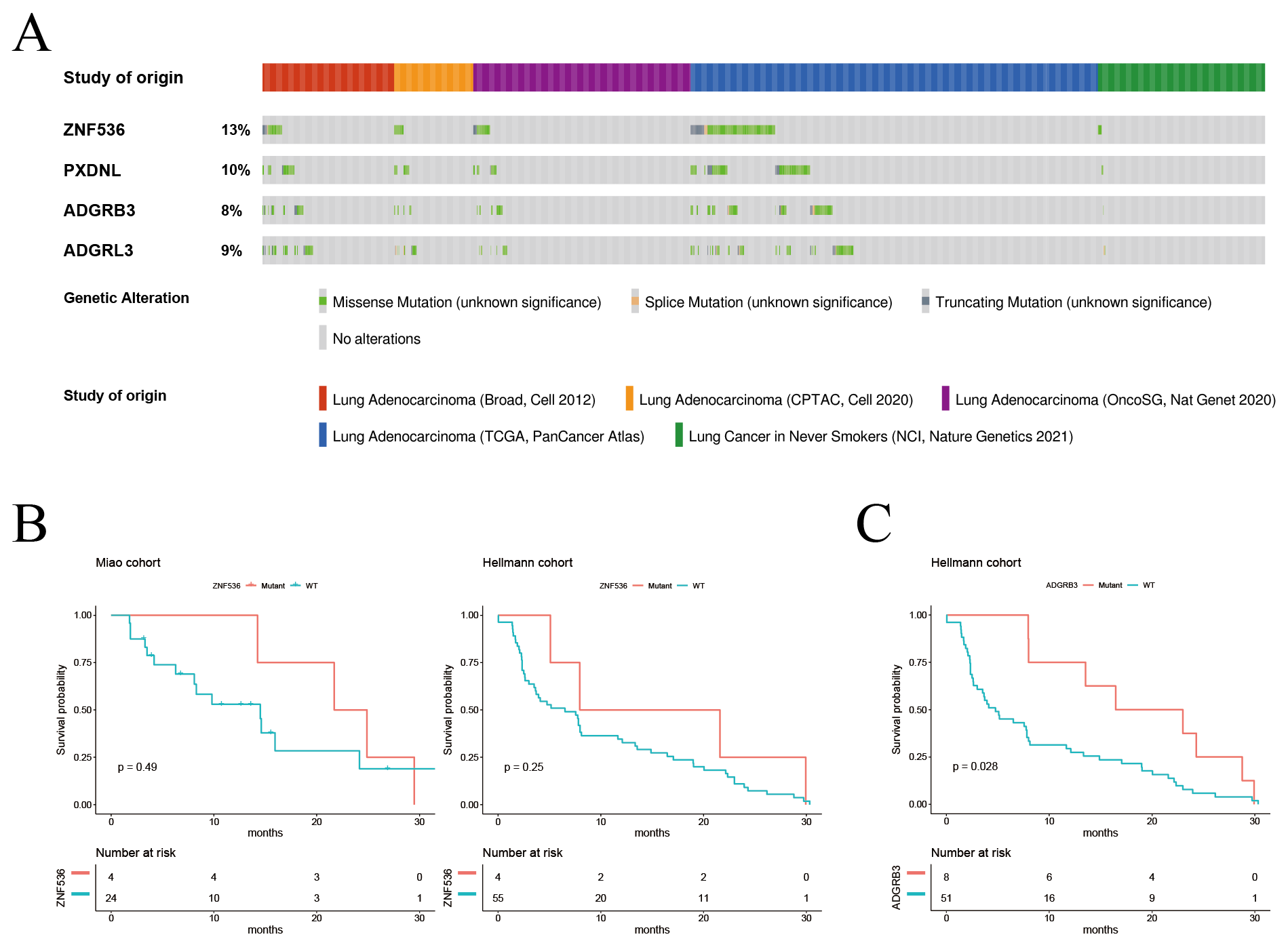


**Figure S4. Prognosis of specific mutations in the immunotherapy cohorts**

(A) Mutation frequencies for ZNF536, PXDNL, ADGRB3, and ADGRL3 in the cBioportal website (https://www.cbioportal.org/). (B) Survival curve of ZNF536 mutation status in Miao's lung adenocarcinoma (n = 28) and Hellmann's nonsquamous NSCLC cohorts (n = 59). (C) Prognostic value of ADGRB3 mutations in the Hellmann's nonsquamous NSCLC cohort (n = 59).


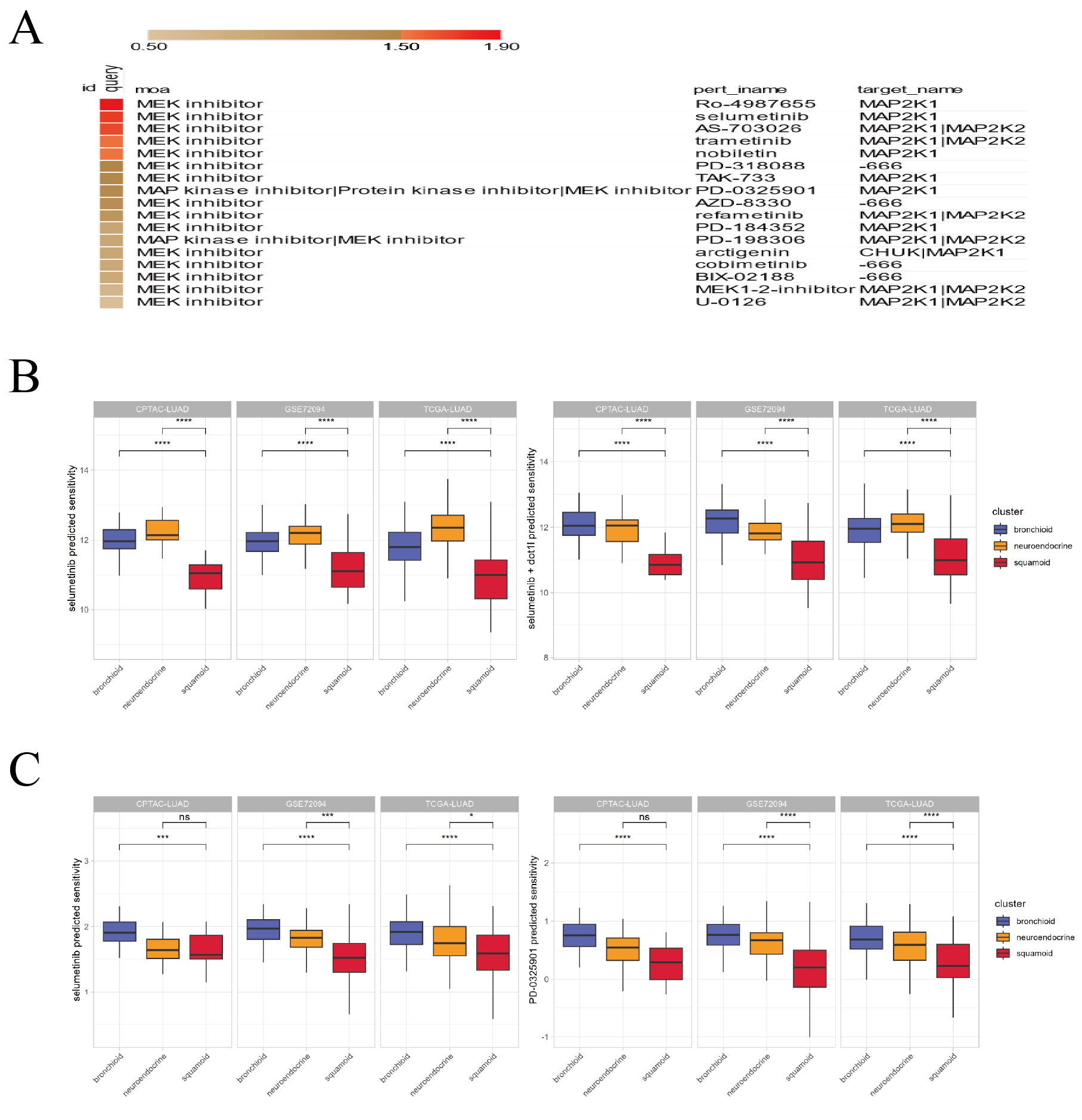


**Figure S5. MEK inhibitors sensitivity predicted by clusters**

1. CMAP enrichment of MEK inhibitors using bronchioid-annotation cell lines. Enrichment greater than absolute 1.5 was considered significant (https://clue.io/morpheus). (B) CCLE predicted sensitivity values in clusters using Wilcoxon test. **** representing p<0.0001 (left: selumetinib; right: selumetinib combination with DOT1L inhibitor). (C) GDSC predicted sensitivity values in clusters using Wilcoxon test. *, *** and **** representing p<0.05, p<0.001, p<0.0001, respectively (left: selumetinib; right: PD-0325901). The values from the GDSC2 version were converted to log_10_ for plotting. Lower values mean therapy sensitivity.


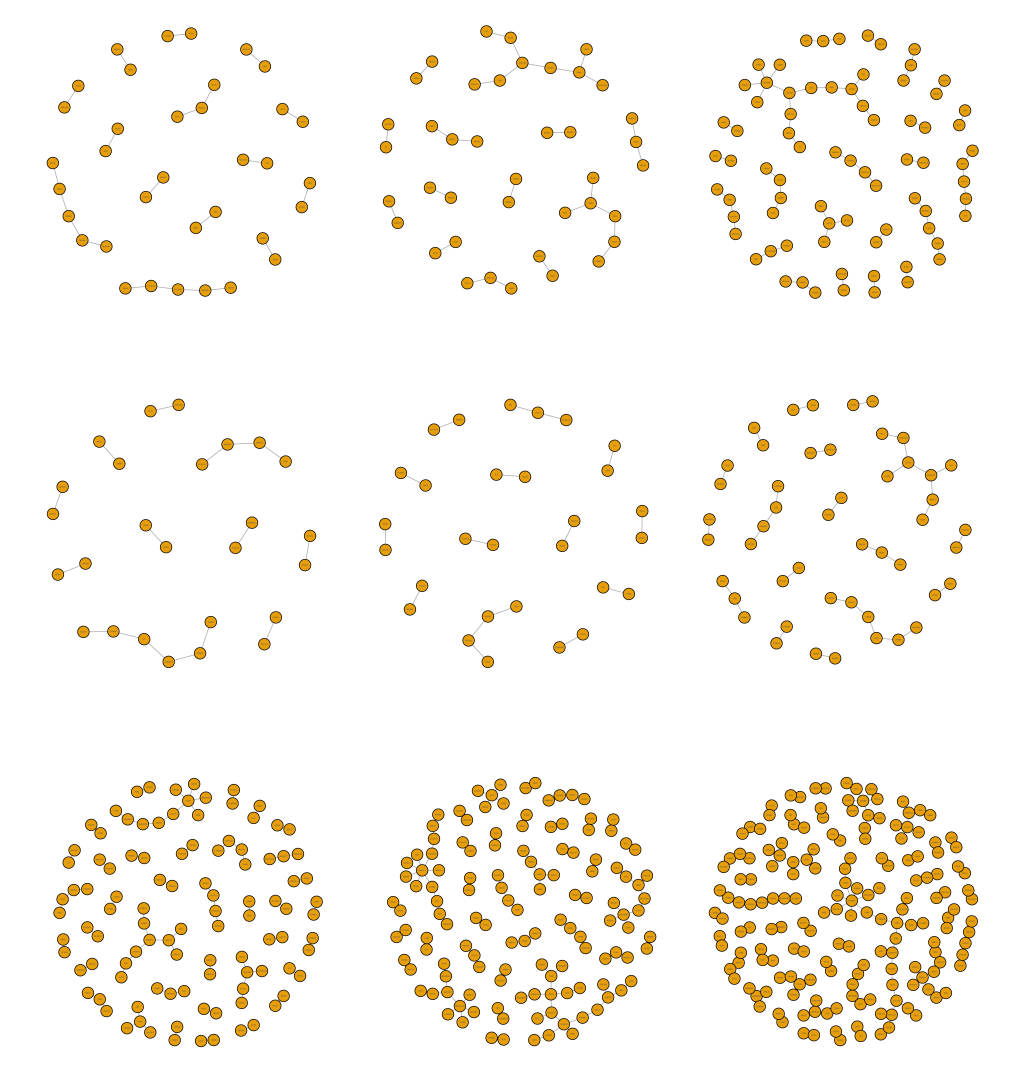


**Figure S6. Clusters-associated differential network**

Constructed Network relied on knockout or overexpression gene information from the CMAP database. Bronchioid, neuroendocrine and squamoid clusters, from top to bottom. From left to right, CPTAC-LUAD, GSE72094, and TCGA-LUAD datasets. Where TCGA-LUAD has the most enriched results.


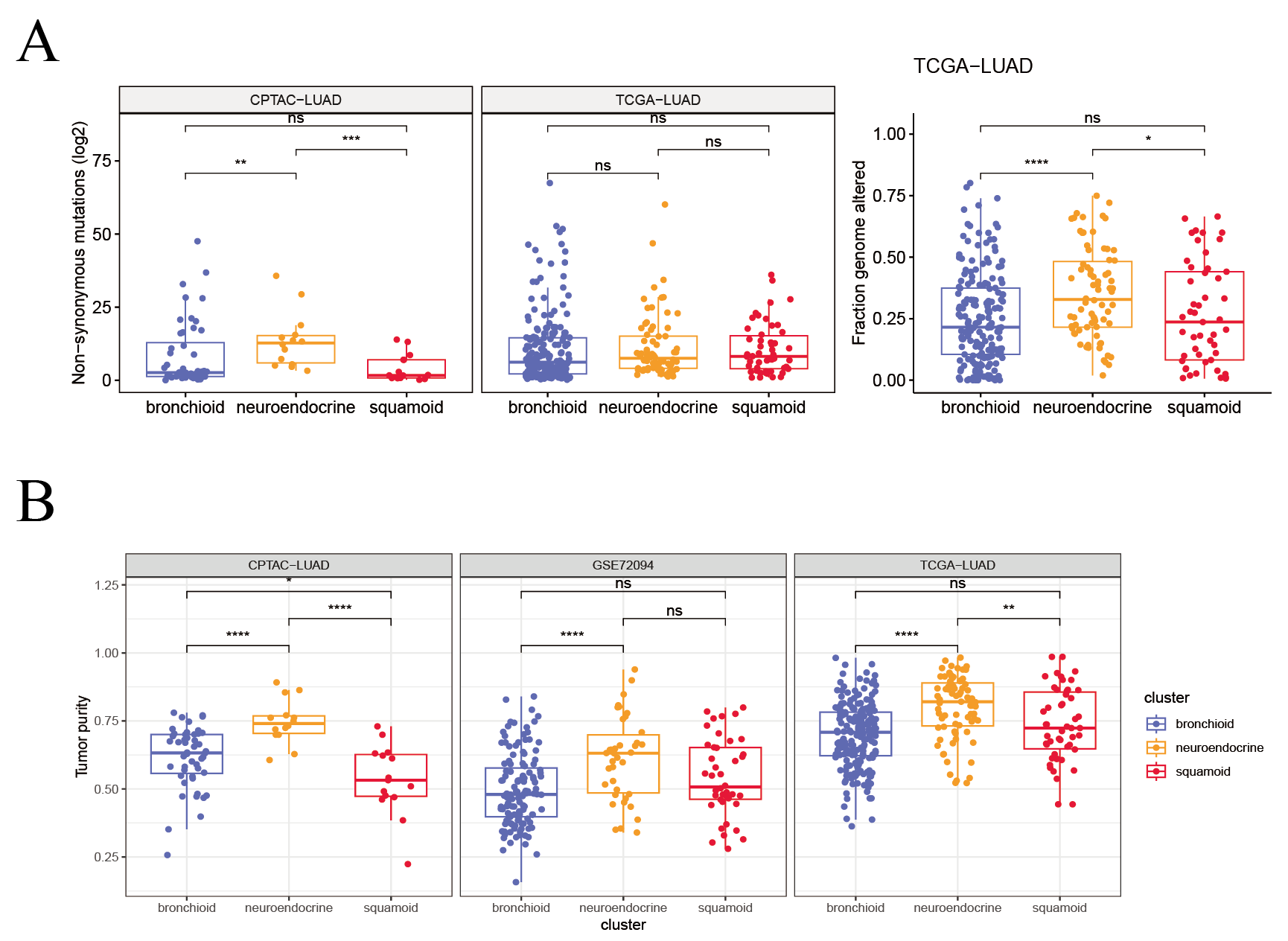


**Figure S7. Tumor burdens, fraction genome altered and tumor purity in clusters**

Boxplot showing (A) Non-synonymous mutations and fraction genome alteration, and (B) tumor purity among three clusters in CPTAC-LUAD, GSE72094, and TCGA-LUAD cohorts using Wilcoxon-test. *, **, *** and **** representing p<0.05, p<0.01, p<0.001, p<0.0001, respectively.


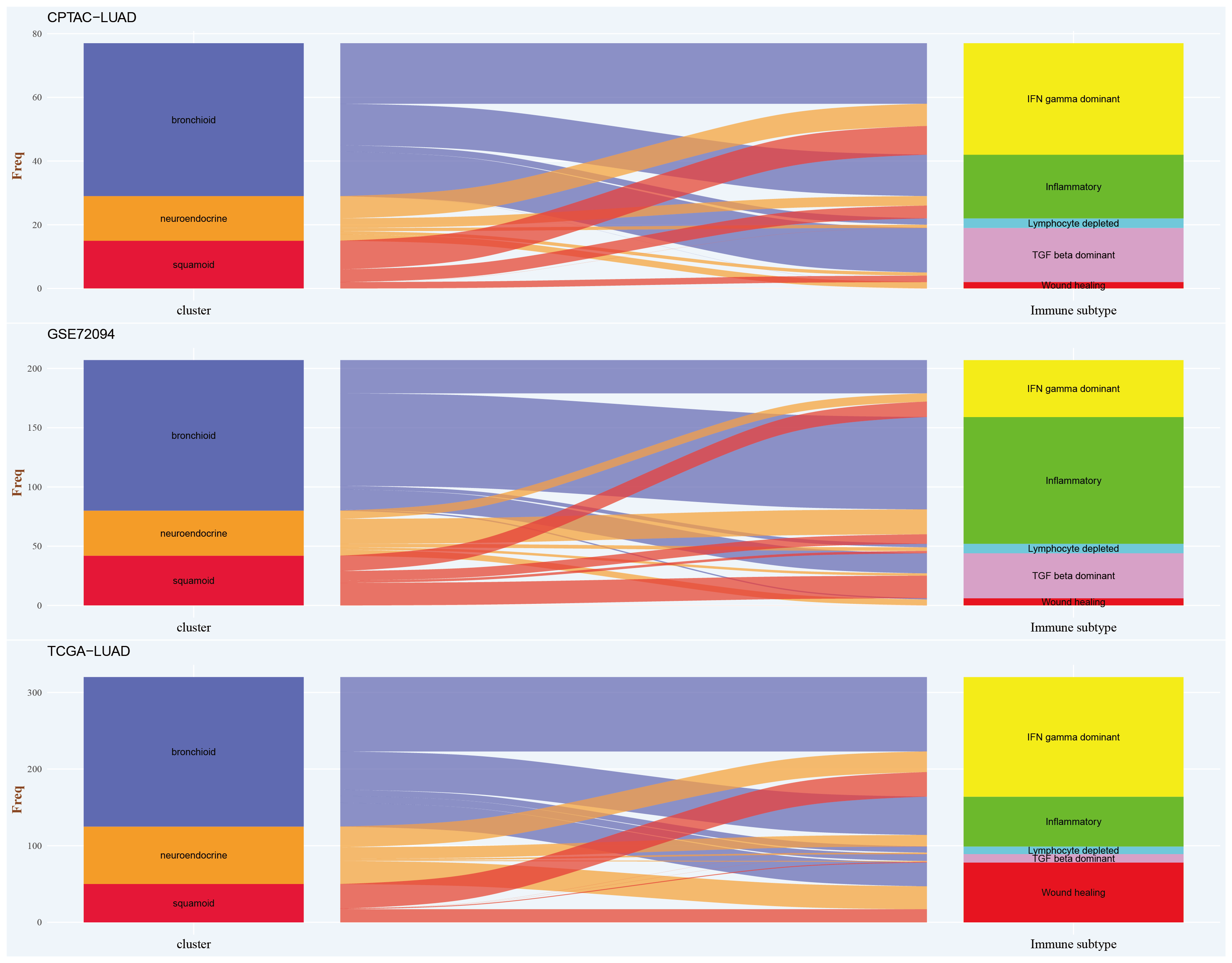


**Figure S8. Association between cluster and classical immunephenotype**

Sankey diagram displaying correspondence between cluster and immunophenotype in CPTAC-LUAD, GSE72094, and TCGA-LUAD cohorts (left: cluster; right: immunophenotype, the Y axes represent the number of tumor samples).


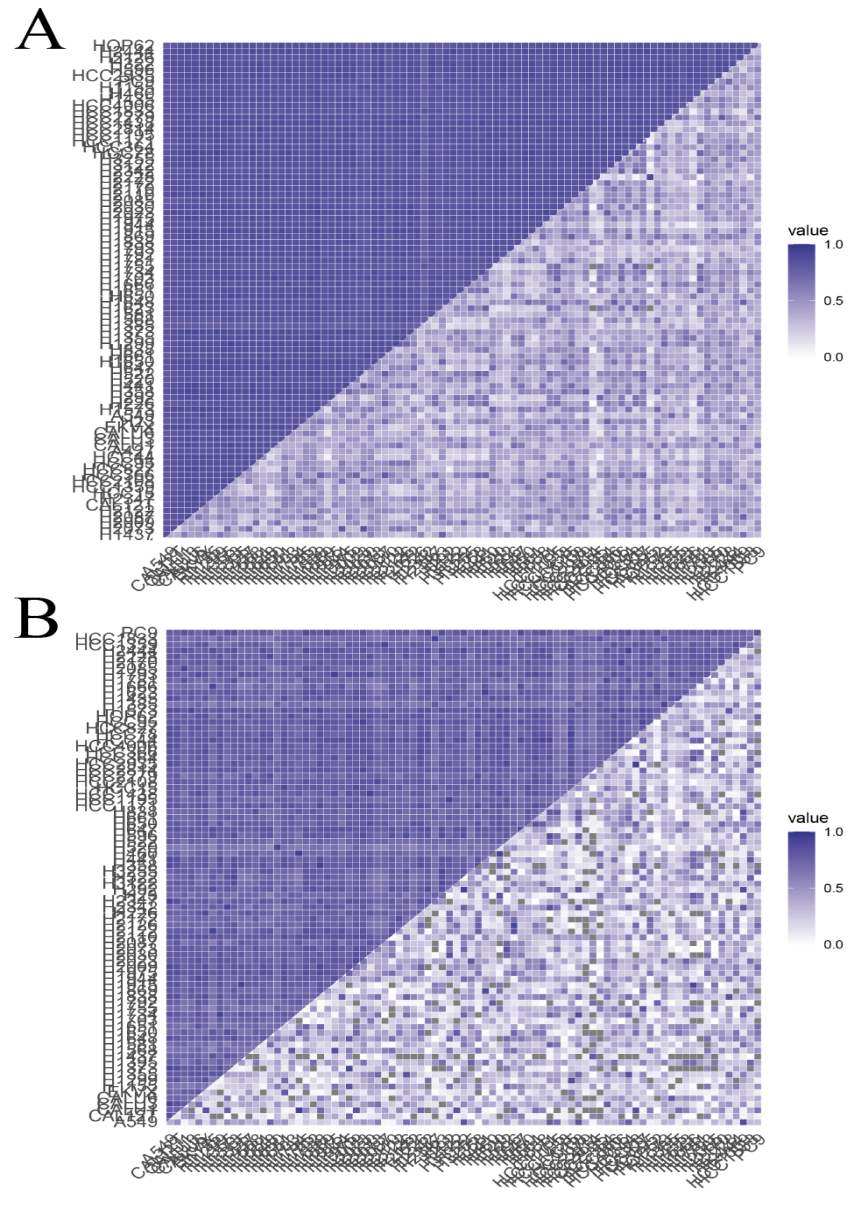


**Figure S9. Validation of cell lines correlations**

1. Correlation matrix comparison using RNA expression (upper triangle: global gene; lower triangle: lineage-specific gene). (B) Correlation matrix comparison using RNA expression (upper triangle: immune pathway; lower triangle: 42-classifier).
